# Bovine Milk Proteome in the First 9 Days: Protein Interactions in Maturation of the Immune and Digestive System of the Newborn

**DOI:** 10.1371/journal.pone.0116710

**Published:** 2015-02-18

**Authors:** Lina Zhang, Sjef Boeren, Jos A. Hageman, Toon van Hooijdonk, Jacques Vervoort, Kasper Hettinga

**Affiliations:** 1 Dairy Science and Technology, Food Quality and Design group, Wageningen University, Wageningen, The Netherlands; 2 Laboratory of Biochemistry, Wageningen University, Wageningen, The Netherlands; 3 Biometris-Applied Statistics, Wageningen University, Wageningen, The Netherlands; 4 Centre for BioSystems Genomics, Wageningen University, Wageningen, The Netherlands; University of Chicago, UNITED STATES

## Abstract

In order to better understand the milk proteome and its changes from colostrum to mature milk, samples taken at seven time points in the first 9 days from 4 individual cows were analyzed using proteomic techniques. Both the similarity in changes from day 0 to day 9 in the quantitative milk proteome, and the differences in specific protein abundance, were observed among four cows. One third of the quantified proteins showed a significant decrease in concentration over the first 9 days after calving, especially in the immune proteins (as much as 40 fold). Three relative high abundant enzymes (XDH, LPL, and RNASE1) and cell division and proliferation protein (CREG1) may be involved in the maturation of the gastro-intestinal tract. In addition, high correlations between proteins involved in complement and blood coagulation cascades illustrates the complex nature of biological interrelationships between milk proteins. The linear decrease of protease inhibitors and proteins involved in innate and adaptive immune system implies a protective role for protease inhibitor against degradation. In conclusion, the results found in this study not only improve our understanding of the role of colostrum in both host defense and development of the newborn calf but also provides guidance for the improvement of infant formula through better understanding of the complex interactions between milk proteins.

## Introduction

Milk is the most important food for the growth and development of the neonate because of its unique nutrient composition combined with the presence of many bioactive components, especially proteins. Human milk is considered as the most suitable food for the infant because it contains proteins which have significant beneficial effects for the babies from both a short-term and a long-term point of view [1]. Although the proteome of bovine milk shows important differences with human milk [2], bovine milk and bovine colostrum have received considerable attention, as they are an important source for the production of ingredients for infant formula and protein supplements [3].

Bovine colostrum contains a wide range of proteins, including high abundant proteins, like αs_1_-casein, αs_2_-casein, β-casein, κ-casein, β-lactoglobulin and α-lactalbumin [4], and low abundant proteins, such as monocyte differentiation antigen CD14 (CD14), glycosylation-dependent cell adhesion molecule 1 (GLYCAM1), xanthine dehydrogenase/oxidase (XDH/XO), lactadherin (MFGE8), and clusterin (CLU) [3]. These proteins not only provide nutrition for the neonates during the initial phase of their lives, but also modulate their immune system to secure healthy growth [5,6]. Apart from the immune function mentioned above, bovine colostrum also contains enzymes involved in digestion, and proteins related to maturation of the neonatal gastrointestinal tract [7–9].

Despite a large number of studies concerning the properties of bovine colostrum, the in-depth study of bovine colostrum proteins was accelerated by the application of proteomic techniques [3]. However, previous proteomic studies mainly focused on the identification of the colostrum proteome [10] and the comparison in the proteome between pooled colostrum and mature milk [3]. No quantitative proteomics studies have been reported that study the change from colostrum to transition milk, using multiple time points from individual cows. A comprehensive understanding of the bovine colostrum proteome and the quantitative changes in time may not only contribute to our knowledge on the needs of the calves, but may also contribute to our understanding of biological functions of milk proteins.

Therefore, the objective of this study is to apply advanced proteomic techniques, the combination of filter aided sample preparation (FASP) and dimethyl labeling followed by LC-MS/MS, to explore the bovine milk serum proteome during the transition from colostrum to milk in the first 9 days after calving. During this period, the low abundant proteins present in colostrum and transition milk will be identified and quantified from four individual cows.

## Materials and Methods

### Materials

Bovine milk was collected from 4 healthy, first-parity, Holstein-Friesian cows from a farm in Zaffelare, Belgium. After the first day, all cows had a somatic cell count lower than 100,000. In order to exclude the influence of diet and management effects, we collected milk from cows on the same farm being managed (including fed) in the same way, with calves born within a short time frame (between 20th August and 27th September 2012). No specific permissions were required for this sample collection, as samples were taken from the milk collected during regular milking. The cows were milked using an automatic milking system, and samples were collected every milking from day 0 to day 9. A total of 100 mL milk was collected at each time point. Samples of each time point were frozen immediately at −20 degree after collection. After finishing sample collection, samples collected after 0, 0.5, 1, 2, 3, 5, 9 days were transferred frozen to the laboratory for further analysis.

### Methods

The methods used in this study are based on two previous articles [2,11].

#### Milk serum separation

The samples collected at each time point of each individual cow were centrifuged at 1500g for 10 minutes at 10°C (Beckman coulter AvantiJ-26 XP centrifuge, rotor JA-25.15). The pellet was removed and the obtained supernatant was transferred to the ultracentrifuge tubes followed by ultracentrifugation at 100.000g for 90 minutes at 30°C (Beckman L-60, rotor 70Ti). After ultracentrifugation, samples were separated into three phases. The top layer was milk fat, the middle layer was milk serum, and the bottom layer (pellet) was casein. Milk serum was used for BCA assay and filter aided sample preparation (FASP) as described below.

#### BCA Assay

BCA Protein Assay Kit 23225 (Thermo Scientific Pierce) was used for protein concentration determination, according to the manufacturer’s instructions. Bovine serum albumin was used as standard for making a calibration curve. The standard curve covers the protein concentration from 0.02–2μg/μL. Subsequently, the milk serum protein concentration was determined.

#### FASP

Milk serum samples (20 μL), including samples of each time point and pooled samples of all the time points from each cow, were diluted in SDT-lysis buffer (100mM Tris/HCl pH 8.0+4% SDS+0.1 M Dithiotreitol) to get a 1 μg/μL protein solution. Samples were then incubated for 10 min at 95°C, and centrifuged at 18407g for 10 min after cooling down to room temperature. 20 μL of sample was directly added to the middle of 180μL 0.05M IAA (Iodoacetamide) /UT (100mM Tris/HCl pH 8.0+8 M urea) in a low binding Eppendorf tube and incubated for 10 min while mildly shaking at room temperature. All of the sample was transferred to a Pall 3K omega filter (10–20 kDa cutoff, OD003C34; Pall, Washington, NY, USA) and centrifuged at 15871g for 30 min. 100 μL of IAA (0.05 M iodoacetamide in UT) was added and incubated for 10 min at room temperature, and then centrifuged at 15871g for 30 min. Three repeated centrifugations at 15871g for 30 min were carried out after adding three times 100 μL UT. After that, 110 μL 0.05 M ABC (0.05 M NH4HCO3 in water) was added to the filter unit and the samples were centrifuged again at 15871g for 30min. Then, the filter was transferred to a new low-binding Eppendorf tube. 100 μL ABC containing 0.5 μg trypsin was added followed by overnight incubation at room temperature. Finally, the sample was centrifuged at 15871g for 30 min, and 3.5 μL 10% trifluoroacetic acid (TFA) was added to the filtrate to adjust the pH value of the sample to around 2. These samples were ready for dimethyl labeling.

#### Dimethyl labeling

The trypsin digested samples of pooled milk serum from each individual cow were labeled with the light reagent (using normal unlabelled formaldehyde and cyanoborohydride), whereas trypsin digested samples of milk serum collected at each time points of each individual cow were labeled with the heavy reagent (using deuterated formaldehyde and normal cyanoborohydride). The dimethyl labeling was carried out according to [12] by on-column dimethyl labelling. Stage tips containing 2 mg Lichroprep C18 (25 um particles) column material (C18+ Stage tip) were made in-house. The C18+ Stage tip column was washed 2 times with 200 μL methanol. The column was conditioned with 100 μL of 1mL/L formic acid (HCOOH) and then samples were loaded on the C18+ Stage tip column. The column was washed with 100 μL 1mL/LHCOOH, and then slowly flushed with 100 μL labeling reagent (0.2% CH_2_O or CD_2_O and 30 mM cyanoborohydride in 50 mM phosphate buffer pH 7.5) in about 10 min. The column was washed again with 200 μL 1mL/L HCOOH. Finally, the labeled peptides were eluted with 50 μL of 70% acetonitrile/30% 1 mL/L HCOOH from the C18+ Stage tip columns. The samples were then dried in a vacuum concentrator (Eppendorf Vacufuge) at 45°C for 20 to 30 minutes until the volume of each sample decreased to 15 μL or less. The pairs of light dimethyl label and heavy dimethyl label were then mixed up and the volume was adjusted to exactly 100 μL by adding 1mL/L HCOOH. These samples were ready for analysis by LC-MS/MS.

#### LC-MS/MS

18 μL of the trypsin digested milk fractions was injected on a 0.10*30 mm Prontosil 300-5-C18H (Bischoff, Germany) pre-concentration column (prepared in house) at a maximum pressure of 270 bar. Peptides were eluted from the pre-concentration column onto a 0.10*200 mm Prontosil 300-3-C18H analytical column with an acetonitrile gradient at a flow of 0.5 μL/min, using gradient elution from 9% to 34% acetonitrile in water with 0.5 v/v% acetic acid in 50 min. The column was washed using an increase in the percentage acetonitrile to 80% (with 20% water and 0.5 v/v% acetic acid in the acetonitrile and the water) in 3 min. A P777 Upchurch micro-cross was positioned between the pre-concentration and analytical column. An electrospray potential of 3.5 kV was applied directly to the eluent via a stainless steel needle fitted into the waste line of the micro-cross. Full scan positive mode FTMS spectra in LTQ-Orbitrap XL (Thermo electron, San Jose, CA, USA) were measured between an m/z of 380 and 1400. CID fragmented MSMS scans of the four most abundant multiply charged peaks in the FTMS scan were recorded in data-dependent mode in the linear trap (MSMS threshold = 5.000).

#### Data analysis

Each run with all MSMS spectra obtained was analysed with Maxquant 1.3.0.5 with Andromeda search engine [13]. A full overview of all MaxQuant parameter is given in S1 Table. Carbamidomethylation of cysteines was set as a fixed modification (enzyme = trypsin, maximally 2 missed cleavages, peptide tolerance 20 ppm, fragment ions tolerance 0.5 amu). Oxidation of methionine, N-terminal acetylation and de-amidation of asparagine or glutamine were set as variable modification for both identification and quantification. The bovine reference database for peptides and protein searches was downloaded as fasta files from Uniprot (http://www.uniprot.org/ accessed March 2012) with reverse sequences generated by Maxquant. A set of 31 protein sequences of common contaminants was added including Trypsin (P00760, bovine), Trypsin (P00761, porcine), Keratin K22E (P35908, human), Keratin K1C9 (P35527, human), Keratin K2C1 (P04264, human), and Keratin K1C1 (P35527, human). A maximum of two missed cleavages were allowed and mass deviation of 0.5 Da was set as limitation for MS/MS peaks and maximally 6 ppm deviation on the peptide m/z during the main search. The false discovery rate (FDR) was set to 1% on both peptide and protein level. The length of peptides was set to at least seven amino acids. Finally, proteins were displayed based on minimally 2 distinct peptides of which at least one unique.

Dimethyl labeling was based on doublets with dimethLys0 and dimethNter0 as light; dimethLys4 and dimethNter4 as heavy labels. Razor and unique peptides were used for quantification. Normalized H/L ratios were used for further statistical analysis. Also the intensity based absolute quantification (iBAQ) algorithm was used in this research. It estimates absolute protein concentration as the sum of all peptide intensities divided by the number of theoretically observable tryptic peptides. The iBAQ value has been reported to have a good correlation with known absolute protein amounts over at least four orders of magnitude [14].

The function of the identified proteins was checked in the UniprotKB database released April 2012 (http://www.uniprot.org/). To select the proteins that significantly decrease over time, proteins were analyzed univariate. For each protein and per cow, a regression line was fitted on the protein concentrations measured at time points 0, 0.5, 1, 2, 3, 5 and 9 days. To reliably estimate a regression line, only proteins with at least 4 observed time points per cow were considered. The regression line summarizes per cow the concentration profiles for each protein into four intercepts and four slopes. The intercepts are the protein concentration at time 0, the slopes indicate the decrease in concentration per day. By using hypothesis tests on the slopes it can be determined if the decrease in concentration is significant. The Lilliefors normality test [15] was used to test if the four slopes were normally distributed. Proteins for which the four slopes were not normally distributed were discarded, since the non-parametric Wilcoxon signed rank test cannot establish a significant decrease with only four observations with α = 0.05. Proteins with normally distributed slopes were subjected to a one-sided t-test to test if the slopes were significantly decreasing (with α = 0.05). Gene Ontology (GO) enrichment analysis was done using DAVID bioinformatics Resources 6.7 [16]. SPSS (Version 21, IBM Corp.) was used to calculate correlation coefficients among quantified proteins. The linear regression and subsequent hypothesis tests between proteins related to complement and coagulation system was performed in Metlab R2012A and Microsoft Excel (2010).

## Results

### Protein concentrations determined by BCA

The protein concentrations of milk serum from four cows collected at different time points are shown in Table 1. There was roughly 10 fold decrease in the protein concentrations from day 0 to day 9 and the rate of change was especially high in the first three days. The total protein content among these four individual cows at day 0 were approximately 2-fold different, whereas the protein content decreased to comparable levels at day 9.

**Table 1:** Protein concentrations determined by BCA assay.

**Time point (day)**	**Protein concentration(μg/μL)**			
	**Cow1**	**Cow2**	**Cow3**	**Cow4**
0	85.28	114.96	141.21	169.55
0.5	53.18	73.78	51.42	78.60
1	22.18	19.44	22.31	29.62
2	14.38	17.02	18.44	20.40
3	12.76	14.20	17.16	19.92
5	12.20	11.26	16.93	20.17
9	15.25	13.39	16.05	15.33

### The number of identified and quantified proteins

**Figure 1:**
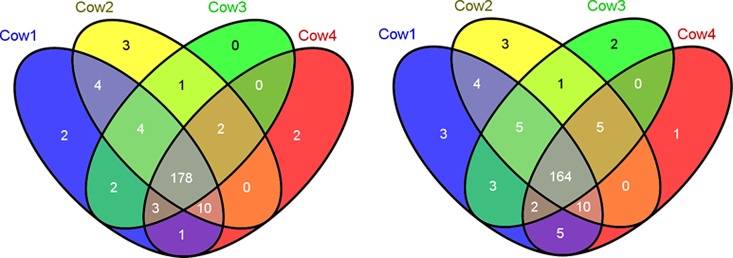
Number of identified (A) and quantified proteins (B) in four biological duplicates.

A total of 212 proteins were identified in all the samples, of which 208 proteins were quantified. In the sample of the four individual cows, around 200 proteins were detected respectively. Of these identified proteins, approximately 98% could be quantified using dimethyl labeling. Moreover, as can be seen in Fig. 1, 80% of identified and quantified proteins were detected in the milk of all individual cows.

### The distribution of biological functions and subcellular locations of identified proteins

**Figure 2:**
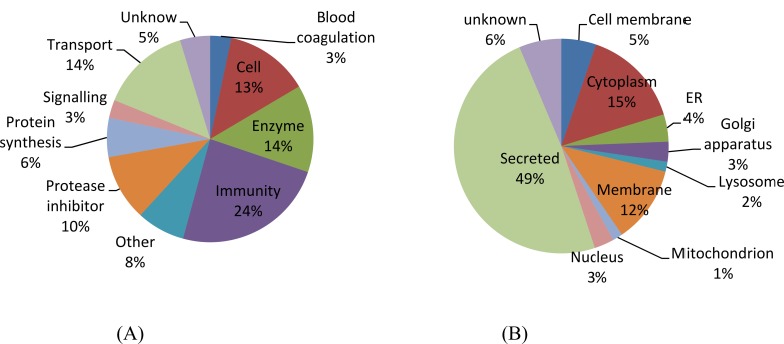
The distribution of biological functions (A) and subcellular location (B) of identified proteins.

The identified proteins were grouped based on their biological function and subcellular location according to Uniprot as shown in Fig. 2. Immune-related protein appeared to be the dominant biological function group (25%). The detailed functions of these immune-related proteins are shown in Table 2 according to the classification of DAVID gene ontology. Enzymes (15%) ranked second, tied with transport proteins (15%). Also, the proportion of protease inhibitors (10%) was relatively high. With respect to subcellular location distribution, 50% of the identified proteins were secreted proteins, followed by cytoplasm (15%) and membrane proteins (12%). Proteins originating from mitochondrion, endoplasmic reticulum (ER), lysosome, Golgi apparatus and nucleus accounted for about 15% in total.

**Table 2:** The number of significant proteins with time-depended changes analysed by t-test.

**Category of immune-related proteins**	**Gene name**
Complement proteins	C1R, C1S, C3, C6, C7, C8B, C9, CFB, CFD, CFH, CFI
Antibacterial proteins	CATHL1, CATHL2, CATHL3, CATHL4, CATHL5, CATHL6, CATHL7, LPO, PGLYRP1
Immunoglobulins-like proteins	A1BG, AHSG, B2M, BoLA, PIGR, IGJ, IGK, IGLL1
Acute phase proteins	ORM1, F2, C3, FN1, SERPINF2, ITIH4, SAA1, SAA3
Other immune-related proteins	AZGP1, B4GALT1, BOLA-NC1, MUC15, CHI3L1, CLU, CRISP3, GLYCAM1, GP2, HP, RNASE4

### The qualitative and quantitative changes of protein between day 0 and day 9 based on biological functions

**Figure 3:**
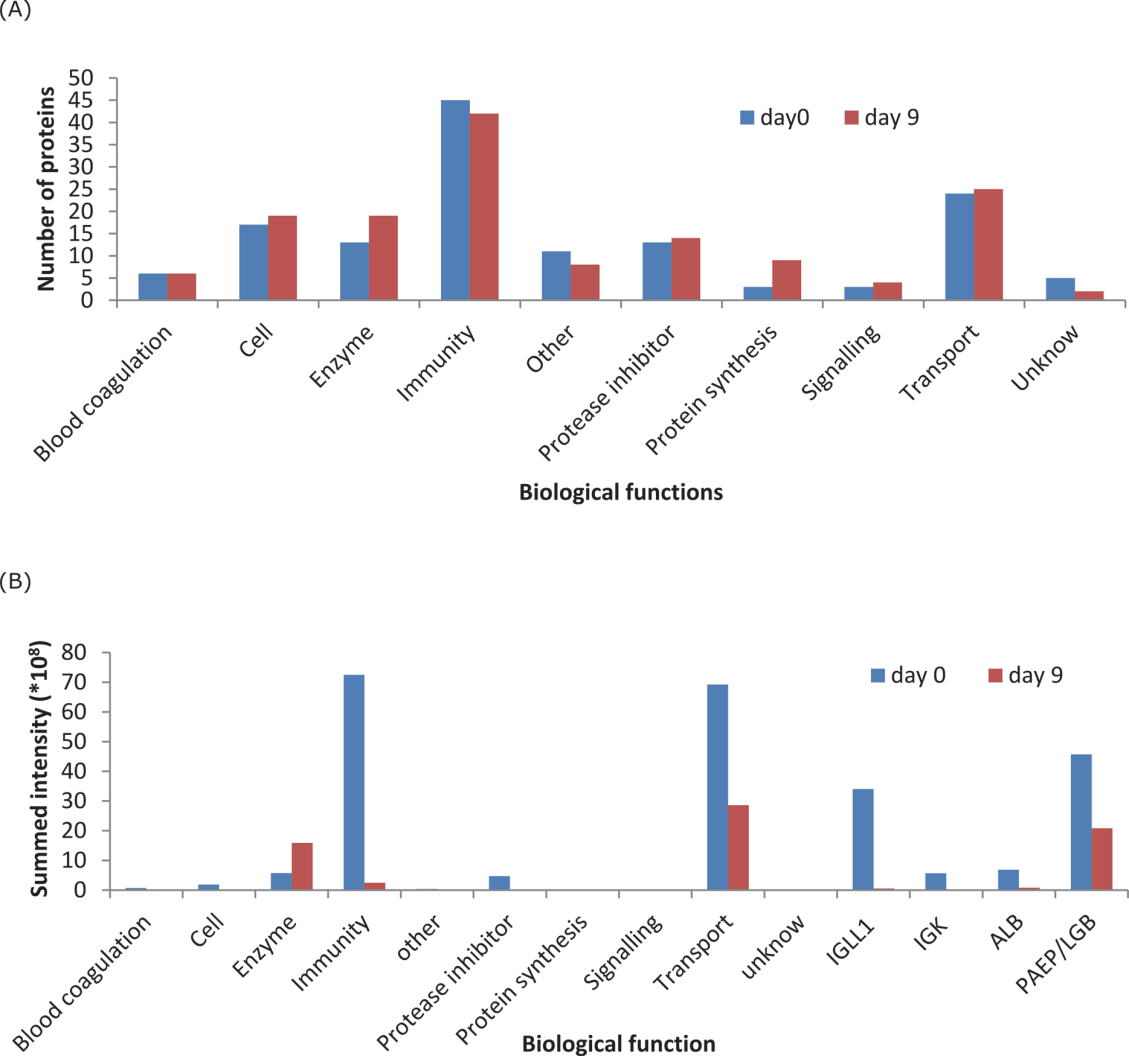
The comparison of biological function distribution of identified proteins (A) and their summed intensities (B) in the milk collected at day 0 and day 9.

The qualitative and quantitative changes of proteins classified by biological function at day 0 and day 9 are shown in Fig. 3. Enzyme is the most different group. Both the number (1.46 fold) and intensity (2 fold) of enzyme show increase from day 0 to day 9. Immune-related proteins showed a slight decrease (10.8%) in the number of identified proteins, and a large decrease (96.6%) in their summed intensities. The total number of transport proteins increased slightly (7.1%) whereas the total summed intensities decreased by 60%. The protease inhibitors showed a different pattern, the number of identified proteins didn’t change from day 0 to day 9 but the intensities decreased remarkably (96.7%); For cell related proteins, the number of identified proteins increased slightly (13%), whilst the intensities decreased remarkably (85%), a similar decrease was also found for the proteins grouped under “others”.

### The quantitative variation of proteins in the milk collected at the first 9 days

**Figure 4:**
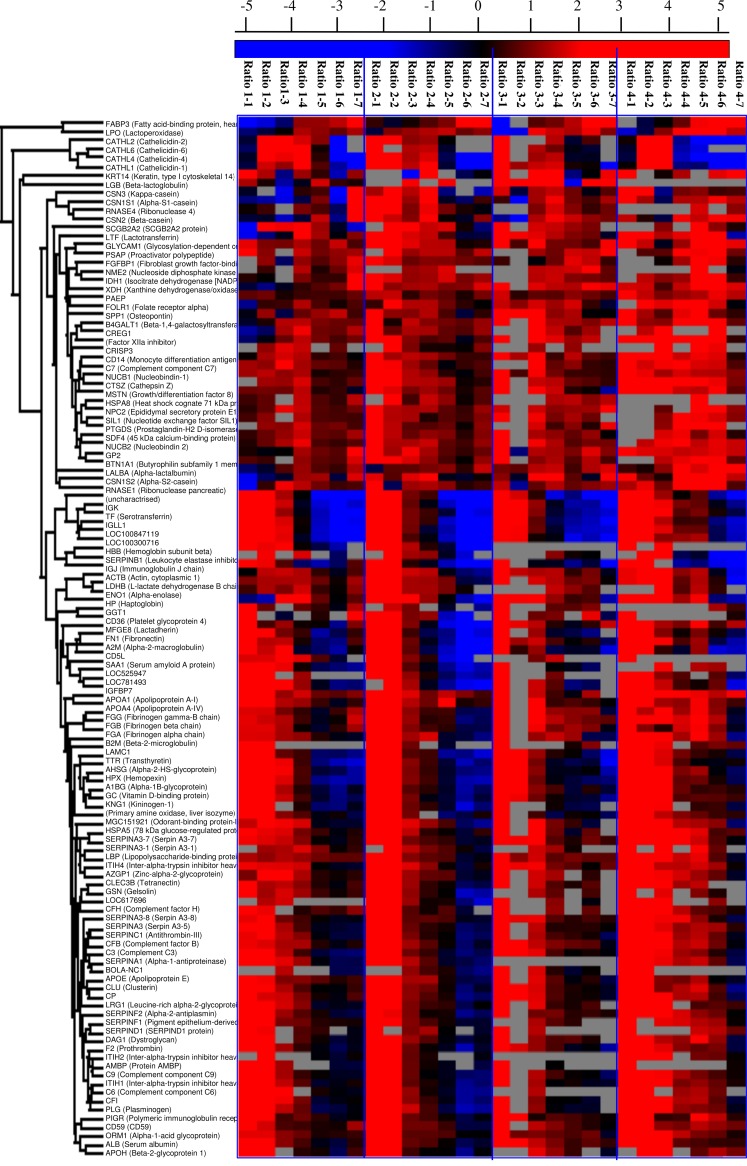
The ratio of identified proteins in the milk collected in the first 9 days with biological duplicates (The red color shows proteins with a log_2_ ratio more than 2, while blue color shows proteins with a log_2_ ratio less than −2. The stronger the color is, the larger the value is. Proteins that couldn’t be quantified are labeled gray.)

The log_2_ ratio of proteins present in at least 14 samples out of 28 samples collected at different time points are shown in a heat map (Fig. 4). The four individual cows show a similar pattern of changes over the first 9 days of lactation. The log_2_ ratio of the majority proteins showed a rapid decrease from day 0 to day 9; A few proteins, however, increased in concentration during the same period (marked with a blue rectangle). This was for instance the case for xanthine dehydrogenase/oxidase (XDH), lipoprotein lipase (LPL), and ribonuclease pancreatic (RNASE1) as shown in Table 3.

**Table 3:** The variation in the average intensities (log10) of four abundant enzymes over the first 9 days.

**Enzyme**	**Day 0**	**Day 0.5**	**Day 1**	**Day 2**	**Day 3**	**Day 5**	**Day 9**
LALBA	8.74	8.74	9.57	9.07	9.03	9.26	9.20
RNASE1	7.10	7.14	7.95	7.49	7.43	7.44	7.07
XDH	6.04	5.71	6.71	6.41	6.34	6.49	6.45
LPL	0.00	4.92	6.31	6.34	4.25	6.14	5.97

**Figure 5:**
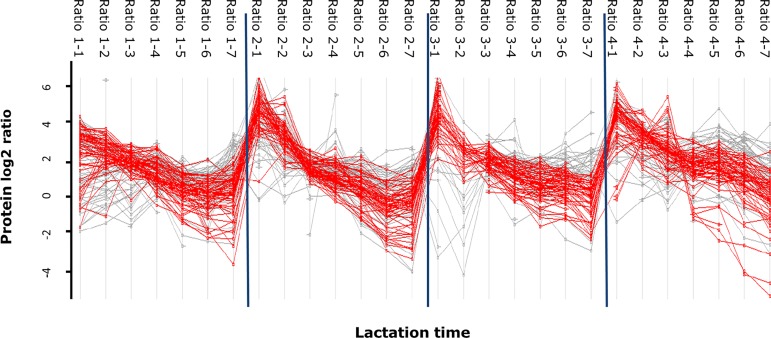
Variation of significant different proteins (one-sided t-test, α = 0.05) in the milk collected in the first 9 days in four individual cows.

In total, 94 proteins were determined at least at four time points per cow. This was deemed as a minimal requirement to reliably estimate the trend over time. The concentration profiles of these 94 proteins were summarized into intercepts and slopes as described in the methods section. The Lilliefors test indicated that 8 proteins had not normally distributed slopes, so these proteins were discarded. From the 86 proteins with normally distributed slopes, a total of 64 proteins showed a significant decrease and they are listed in Table 4. For some individual proteins, the decrease in concentration from day 0 to day 9 was as high as 40 fold. The changes in protein ratios over time of quantified proteins (grey) and the significantly different proteins (red) are shown in Fig. 5. For instance, IGJ, decreased by 65% in the first 12 hours and 85% after 1 day; IGK by 32% in the first 12 hours and 69% after 1 day. SERPINA1, GSN, ITIH1 decreased by 88%; PLG by 95% and KNG1 by 93% after three days as shown in S2 Table. Although the four individual cows showed the same pattern in the reduction of low abundant proteins, the concentration of certain proteins at day 0 could differ as much as 5 fold. The concentrations of most proteins in the milk of cow 1 were lower compared to the other three cows, which agrees with the BCA results as shown in Table 1.

**Table 4:** Significant different proteins with time series (one-sided t-test, α = 0.05).

**Protein IDs**	**Protein name**	**Gene name**	**Biological function**	**Subcellular location**	**p value**
P01044	Kininogen-1	KNG1	Blood coagulation	secreted	0.000
P02672	Fibrinogen alpha chain	FGA	Blood coagulation	secreted	0.004
P02676	Fibrinogen beta chain	FGB	Blood coagulation	secreted	0.014
P06868	Plasminogen	PLG	Blood coagulation	secreted	0.009
P12799	Fibrinogen gamma-B chain	FGG	Blood coagulation	secreted	0.000
P17690	Beta-2-glycoprotein 1	APOH	Blood coagulation	secreted	0.006
P02769	Serum albumin	ALB	Cell	secreted	0.013
Q2KIF2	Leucine-rich alpha-2-glycoprotein 1	LRG1	Cell	secreted	0.005
F1N076		CP	Cell	secreted	0.003
F1MPP2		IGFBP7	Cell adhension	secreted	0.014
P31096	Osteopontin	SPP1	Cell adhension	secreted	0.037
F1N514		CD5L	Cell apootosis	membrane	0.002
P02702	Folate receptor alpha	FOLR1	Cell death	cell membrane	0.042
O18738	Dystroglycan	DAG1	Cell-Cytoskeleton	secreted	0.000
F1N4M7		CFI	Enzyme	membrane	0.020
P05689	Cathepsin Z	CTSZ	Enzyme	Lysosome	0.001
Q29437	Primary amine oxidase, liver isozyme		Enzyme	secreted	0.016
Q5E9B1	L-lactate dehydrogenase B chain	LDHB	Enzyme	secreted	0.013
F1MZ96		IGK	Immunity	secreted	0.001
P00735	Prothrombin	F2	Immunity	secreted	0.000
P01888	Beta-2-microglobulin	B2M	Immunity	secreted	0.000
P07589	Fibronectin	FN1	Immunity	secreted	0.019
P12763	Alpha-2-HS-glycoprotein	AHSG	Immunity	secreted	0.021
P17697	Clusterin	CLU	Immunity	secreted	0.002
P19660	Cathelicidin-2	CATHL2	Immunity	secreted	0.011
P22226	Cathelicidin-1	CATHL1	Immunity	secreted	0.003
P28800	Alpha-2-antiplasmin	SERPINF2	Immunity	secreted	0.002
P33046	Cathelicidin-4	CATHL4	Immunity	secreted	0.001
P54228	Cathelicidin-6	CATHL6	Immunity	secreted	0.005
P81187	Complement factor B	CFB	Immunity	secreted	0.002
P81265	Polymeric immunoglobulin receptor	PIGR	Immunity	secreted	0.001
Q29RQ1	Complement component C7	C7	Immunity	secreted	0.010
Q2KJF1	Alpha-1B-glycoprotein	A1BG	Immunity	secreted	0.000
Q2TBU0	Haptoglobin	HP	Immunity	secreted	0.023
Q2UVX4	Complement C3	C3	Immunity	secreted	0.001
Q32PA1	CD59	CD59	Immunity	membrane	0.017
Q3MHN2	Complement component C9	C9	Immunity	secreted	0.007
Q3SYR8	Immunoglobulin J chain	IGJ	Immunity	secreted	0.012
Q3SZR3	Alpha-1-acid glycoprotein	ORM1	Immunity	secreted	0.002
Q3T052	Inter-alpha-trypsin inhibitor heavy chain H4	ITIH4	Immunity	secreted	0.012
Q3ZCH5	Zinc-alpha-2-glycoprotein	AZGP1	Immunity	secreted	0.001
Q7SIH1	Alpha-2-macroglobulin	A2M	Immunity	secreted	0.004
Q95122	Monocyte differentiation antigen CD14	CD14	Immunity	cell membrane	0.001
Q0P569	Nucleobindin-1	NUCB1	Other	Golgi apparatus	0.002
Q3SX14	Gelsolin	GSN	Other	Cytoplasm	0.015
Q3ZBZ1	45 kDa calcium-binding protein	SDF4	Other	Golgi apparatus	0.034
A2I7N1	Serpin A3–5	SERPINA3	Protease inhibitor	cytoplasm	0.002
F1MSZ6	Antithrombin-III	SERPINC1	Protease inhibitor	secreted	0.001
P34955	Alpha-1-antiproteinase	SERPINA1	Protease inhibitor	secreted	0.000
Q0VCM5	Inter-alpha-trypsin inhibitor heavy chain H1	ITIH1	Protease inhibitor	secreted	0.005
Q9TTE1	Serpin A3–1	SERPINA3–1	Protease inhibitor	cytoplasm	0.001
P60712	Actin, cytoplasmic 1	ACTB	Protein synthesis	Cytoplasm	0.010
Q0VCX2	78 kDa glucose-regulated protein	HSPA5	Protein synthesis	ER	0.025
A6QPK0	SCGB2A2 protein	SCGB2A2	Signalling	secreted	0.030
O46375	Transthyretin	TTR	Transport	secreted	0.009
P15497	Apolipoprotein A-I	APOA1	Transport	secreted	0.017
Q03247	Apolipoprotein E	APOE	Transport	secreted	0.008
Q0IIA2	Odorant-binding protein-like	MGC151921	Transport	secreted	0.006
Q29443	Serotransferrin	TF	Transport	secreted	0.000
Q32KV6	Nucleotide exchange factor SIL1	SIL1	Transport	ER	0.032
Q3MHN5	Vitamin D-binding protein	GC	Transport	secreted	0.000
Q3SZV7	Hemopexin	HPX	Transport	secreted	0.000
F1MLW8		LOC100847119	unknown	unknown	0.000
G3N1R1		LOC100300716	unknown	unknown	0.000

*Note: ER is endoplasmic reticulum

### Correlation analysis of proteins

**Figure 6:**
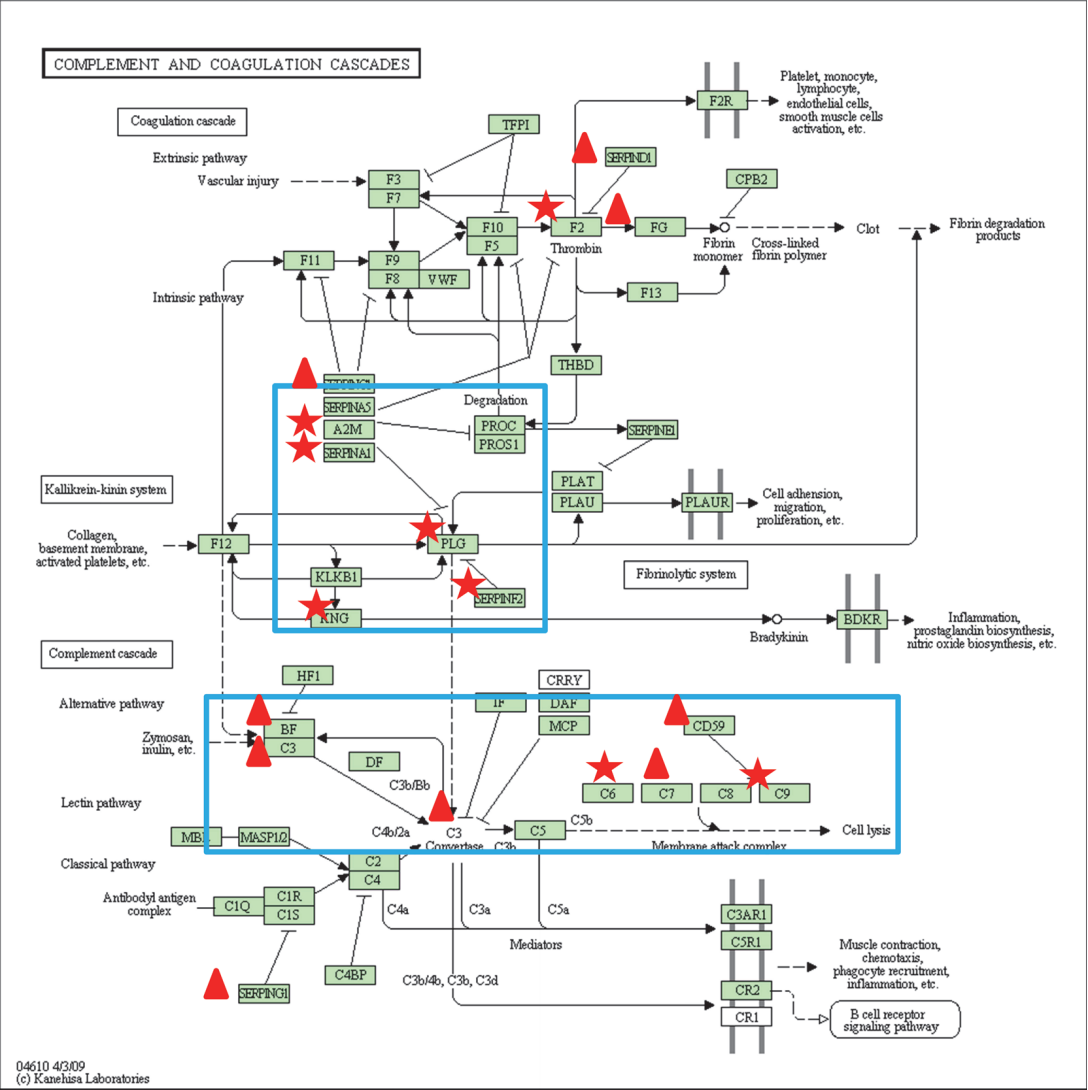
Significant different proteins involved in complement and coagulation cascades.

**Figure 7:**
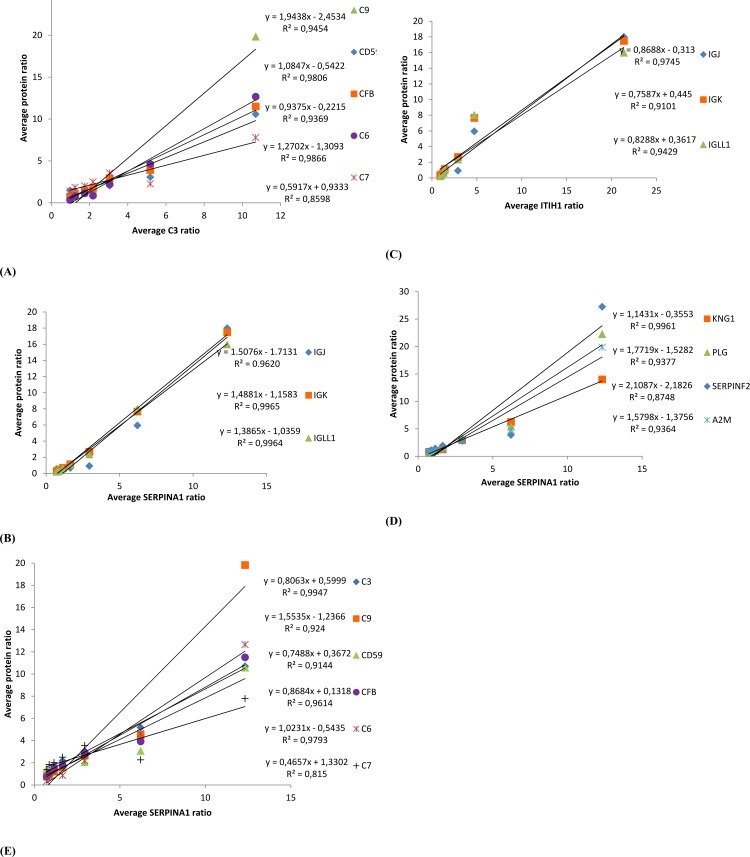
The correlation of significantly different proteins involved in complement, coagulation pathway, and immunoglobulins.

Correlation analysis was also conducted among proteins (140), which can be identified and quantified in at least half of the samples. Several immune-related proteins, coagulation-related proteins and protease inhibitors were found to be highly correlated (r^2^ >0.80) as shown in S3 Table. Based on the DAVID gene ontology analysis, we found that most of the highly correlated proteins participate in the complement system and coagulation cascade as shown in Fig. 6. The significantly different proteins are mainly related to two specific parts of these pathways, the complement cascade and the kallikrein-kinin system. These two pathways were reported to have many similarities because both cascades utilize multi-domain serine proteases with a similar domain structure as catalysts. Correlation coefficients of proteins involved in these two specific pathways (SERPINA1, A2M, PLG, KNG1, C3, C6, C7, C9, CFB, CD59 and SERPINF2) were also between 0.815 to 0.997 as shown in Fig. 7A, 7D, and 7E). In addition, protease inhibitors were found to be highly correlated with complement proteins and immunoglobulins (S3 Table). Two examples (SERPINA1 and ITIH1) are shown in Fig. 7B and 7C.

## Discussion

### Overview of the whole proteome

This study investigated the time-dependent changes of low abundant proteins in bovine milk in the first 9 days of lactation, using dimethyl labeling for quantification. For optimal quantification results, we mixed the sample collected at each time point labelled with heavy reagent with a single pooled sample labelled with light reagent as reference. All analyses were thus done relative to the same reference. A total of 212 proteins were identified in bovine milk sample, of which 208 proteins were quantified. Most of the low abundant proteins reported in proteomics studies of bovine colostrum and milk [6,17–19] were also found to be present in the current study. In addition, we also identified and quantified many proteins, as shown in S2 Table, which have not been reported by Zhang, et al. (2011) who used 2-D gel separation combined with LC-MS/MS [20]. The relatively higher number of identified and quantified proteins found in this study confirms the suitability of FASP and dimethyl labelling combined with LC-MS/MS on identifying and quantifying low abundant proteins in milk [11]. The proteins, identified or quantified for the first time in the current study, were often identified in only a few time points in individual cows. This may be due to the sensitivity of the instrument used in this study because the concentration of these proteins were all around the instrument detection limit. The overlap between the four individual cows in both identified (83% found in all four cows) and quantified proteins (78%) gives an indication of the similarity of the milk proteome among individual cows. In addition, all four cows showed similar changes in protein concentrations over time as shown in Table 1 and Fig. 5. Approximately one-third of all proteins changed significantly (*p*<0.05; up to 40 fold) over the first 9 days as shown in Table 4. At the same time, there were also differences in protein concentrations between individual cows. These differences may be caused by individual differences as a more than 20-fold difference in concentration was found to have within a herd of 189 healthy cows [6].

The classification of identified proteins as shown in the Fig. 2 (A) depicts the different biological functions of the bovine milk proteome. Based on the comparison of identified proteins and their intensities between day 0 and day 9 (Fig. 3A and 3B), we may conclude that the variation in the milk proteome is determined by concentration rather than by composition. The remarkable decrease in the summed intensities of immune-related protein was mainly attributed to immunoglobulins (IGJ and IGK), which will be discussed further below. The large decrease in the summed intensities of transport proteins was mainly caused by the decrease of the major proteins β-lactoglobulin (LGB) and serum albumin(ALB), as shown in Fig. 3 (B). The rapid decrease of LGB and ALB in the first few days is in agreement with the results of previous studies[21].

### Proteins involved in the development of the gastro-intestinal tract

Next to transport proteins, enzymes also showed an increase in intensities from day 0 to day 9. The increase in intensities is attributed to four high abundant enzymes, which contributed to LALBA, XDH, LPL, and RNASE1 (Table 3). LALBA regulates subunit of lactose synthase [22], but it doesn’t have a catalytic activity. The up-regulation of XDH was previously reported for bovine milk serum [3] during the transition from colostrum to mature milk. This increase may be related to mitigation of the oxidative stress in newborns, because it exerts an antimicrobial activity through inducing reactive oxygen species (ROS) generation [23]. XDH may function in the digestion system of calves as it has been reported to play a key role in blood-meal digestion in flies [24]. LPL is an enzyme that is secreted from the pancreas into the digestive tract but also transferred from the lactating mammary gland into the milk [25]. In the gastrointestinal tract, bovine LPL functions in digestion of triglycerides and absorption of lipid nutrients in newborns [26]. RNASE1, is another pancreatic enzyme that plays a major role in digestion of nucleic acids of microorganisms in the lumen of calves [27]. RNASE1 has been thought to play a role in the nutrient uptake in the gut and in the degrading of bacterial RNA in the intestinal tract [28], which is especially important in plant eating animals like cows.

Although digestive functions of calves develop during fetal life, the gastrointestinal tract cannot be fully developed in 2–3 days after birth, and this development continues until the calve fully transitioned to solid food [29]. As the intestine and the pancreas of calves are not mature at the age of 7 days [27], the digestion process probably depends also on the enzymes transferred from colostrum or milk to the calf. The increase of XDH, LPL, RNASE1 over the first 9 days suggests the important roles in the digestion processes of the newborn calf.

Whereas the enzymes involved in digestion increased in the first 9 days, protein related to development of the gastrointestinal tract showed a decrease. Cell division and proliferation protein CREG1, a secreted glycoprotein, has been reported as cellular repressor inhibiting cell proliferation and enhancing cell differentiation in human embryonic carcinoma cells [30]. Growth cytokine MSTN is a member of the transforming growth factor (TGF)-β family, which is one of the predominant growth factors present in bovine milk [31]. These growth cytokines have been reported to promote the growth and development human intestine [32]. This is to be expected, as colostrum and milk has been reported to provide proteins related to maturation of the neonatal gastrointestinal tract [7–9].

### Proteins involved in development of the immune system

Based on biological functions the dominant groups of proteins are the immune-related proteins, for which both the number and summed intensities decreased considerably over the first 9 days. The decrease of intensities is mainly driven by a decrease of the immunoglobulins as is shown in Fig. 3(B). Immunoglobulins showed a high abundance in the first two days and then decreased steeply afterwards. Also other immune-related proteins such as A2M, C9, A1BG, AHSG, CLU, decreased significantly during these 9 days as shown in S2 Table. A decrease in immune-related proteins in bovine milk has been reported before by Le, Barton et al. (2011), Senda, Fukuda, et al. (2011), and Zhang, et al. (2011) [3,19,20]. The relative higher concentration of immune-related proteins in colostrum compared to mature milk was also reported in yak milk [33]. The high concentration of immunoglobulins in the first two days is mainly due to the important role in the build-up of the adaptive immune system of calves, since they don’t get any immunoglobulins from the mother cow before they are born [34]. The significant decrease of immune-related proteins in the first 9 days is probably related to the decrease in the ability to transfer immune-related proteins from cows to calves [35]. This transfer during the first two days has been linked to the immature digestion system of calves, the high pH of abomasum content, and the relatively low pH in the proximal duodenum, which are all favorable conditions to reduce enzyme action and thus allow sufficient absorption of intact immune-related proteins during the first days of live [27]. After the fast development of the calves’ immune system during the first two days, the reliance of calves on immune proteins from colostrum also reduces [36].

In addition to these major proteins of the adaptive immune system, we also found a range of complement components (A2M, C3, C6, C7, C9, CFB, and CD59) that are important for the innate immune system (Fig. 6). These components are also present in high concentrations in the first two days and decrease at comparable rates during the following days (Fig. 7A). CD59 is one of the main regulators of the complement pathway [37], which limits C9 input and prevents the polymerization of C9 during the final step of membrane attack complex (MAC) formation on the cell membrane [38]. When the cow is infected by pathogens, the level of CD59 drops and therefore its inhibitory role in the complement system will be reduced, thereby allowing the complement system to function during the inflammation [39]. The high abundance of complement proteins indicates that colostrum not only confers components from the adaptive immune system to the newborns but that it also transfers proteins of the innate immune system [40].

Although protease inhibitors have been previously reported to be present in milk, their potential function in milk is still unclear. In this study, we not only found high abundant protease inhibitors but also found a high correlation of the protease inhibitors with other immune-related proteins (S3 Table). Several protease inhibitors, such as SERPINA1, GSN, AMBP, ITIH1, ITIH2, decreased significantly (Table 4). The decrease of these protease inhibitors was highly correlated with a similar decrease in immunoglobulins. Two examples are shown in Fig. 7B and 7C. The high correlation between protease inhibitors and immunoglobulins agrees with a previous study which reported that SERPINA1 can protect IgG [41] and lactoferrin [42] from proteolytic degradation. Therefore, protease inhibitors in milk may help protecting immune-related proteins. The same reduction of protease inhibitors and immunoglobulins may also be caused by protein-protein interactions within complex [43]. In addition, protease inhibitors are also involved in the blood coagulation cascade and complement pathway [44] as shown in Fig. 6. The participation of proteases and protease inhibitors in the immune response and blood coagulation [45] can be attributed to the complexity and interactions of milk proteins in biological functions. As an example, the activity of PLG, which is a protease that functions as blood coagulation protein, has also been shown to increase during severe mastitis [46]. PLG can act as a cofactor in adhesion, or, following activation to plasmin, provide a source of potent proteolytic activity of bacterial cells [47]. Proteins A2M, AHSG, C3, ITIH4, SERPINF2, which are classified as immune-related proteins, also function as protease inhibitor according to DAVID Gene Ontology [16], whereas complement pathway proteins F2, C1S, CFB, CFD, HP, have serine-type endopeptidase activity [16]. SERPINA1 has been previously reported to regulate leucocyte-released serpin proteinase activity during complement activation and inflammation, and it was also shown to be involved in the blood coagulation system through inhibiting coagulation pathway enzymes [48]. These proteins related to the complement and coagulation pathways were also reported in yak milk [33]. The correlated changes of immune-related proteins, protease inhibitors and blood coagulation proteins (Fig. 7D and 7E) agrees with the result from previous studies [49,50]. This phenomenon is probably related to the balance between proteases and protease inhibitors that are involved in not only blood clotting, but also cytokine activation and inflammation.

In conclusion, this study for the first time shows the quantitative changes of the milk proteome from four individual cows at 7 time points between day 0 and day 9. Non-targeted proteomics analysis combined with time series study contributes to our understanding of the needs of the calf in the first days of life, as well as the complex biological interactions of milk proteins in the growth and development of newborns. This study also indicates which proteins may be of importance to the newborn and therefore warrant further targeted investigations.

## Supporting Information

S1 Table All parameters used in the MaxQuant software. (XLSX)

S2 Table Full data of the identified and quantified proteins in all samples. (XLSX)

S3 Table Correlation coefficients between all proteins identified. (XLSX)
